# Identification of key genes and pathways of BMP-9-induced osteogenic differentiation of mesenchymal stem cells by integrated bioinformatics analysis

**DOI:** 10.1186/s13018-021-02390-w

**Published:** 2021-04-20

**Authors:** Jia-qi Wu, Lin-bo Mao, Ling-feng Liu, Yong-mei Li, Jian Wu, Jiao Yao, Feng-huan Zhang, Ting-yu Liu, Ling Yuan

**Affiliations:** 1 Rehabilitation Department, Jingjiang People’s Hospital, No.28, Zhongzhou road, Jingjiang, Taizhou, 214500 Jiangsu Province China; 2 Institute Office, Jingjiang People’s Hospital, Jingjiang, China

**Keywords:** Differentially expressed genes, BMP-9, Mesenchymal stem cells, Enrichment analysis

## Abstract

**Background:**

The purpose of present study was to identify the differentially expressed genes (DEGs) associated with BMP-9-induced osteogenic differentiation of mesenchymal stem cells (MSCs) by using bioinformatics methods.

**Methods:**

Gene expression profiles of BMP-9-induced MSCs were compared between with GFP-induced MSCs and BMP-9-induced MSCs. GSE48882 containing two groups of gene expression profiles, 3 GFP-induced MSC samples and 3 from BMP-9-induced MSCs, was downloaded from the Gene Expression Omnibus (GEO) database. Then, DEGs were clustered based on functions and signaling pathways with significant enrichment analysis. Pathway enrichment analysis using the Kyoto Encyclopedia of Genes and Genomes (KEGG) demonstrated that the identified DEGs were potentially involved in cytoplasm, nucleus, and extracellular exosome signaling pathway.

**Results:**

A total of 1967 DEGs (1029 upregulated and 938 downregulated) were identified from GSE48882 datasets. R/Bioconductor package limma was used to identify the DEGs. Further analysis revealed that there were 35 common DEGs observed between the samples. GO function and KEGG pathway enrichment analysis, among which endoplasmic reticulum, protein export, RNA transport, and apoptosis was the most significant dysregulated pathway. The result of protein-protein interaction (PPI) network modules demonstrated that the Hspa5, P4hb, Sec61a1, Smarca2, Pdia3, Dnajc3, Hyou1, Smad7, Derl1, and Surf4 were the high-degree hub nodes.

**Conclusion:**

Taken above, using integrated bioinformatical analysis, we have identified DEGs candidate genes and pathways in BMP-9 induced MSCs, which could improve our understanding of the key genes and pathways for BMP-9-induced osteogenic of MSCs.

## Introduction

Mesenchymal stem cells (MSCs) are non-hematopoietic multipotent cells and used for bone tissue regeneration [1, 2]. MSCs can differentiate into osteoblastic, chondrogenic, and other lineages through different stimulating factors [3, 4]. Bone morphogenetic proteins (BMPs) are a group of transforming growth factor-β (TGF-β) and play an important role in embryonic development, growth, and differentiation [5]. BMP-9 (also known as growth/differentiation factor-2) has been shown to play a pivotal role in many physiological processes including neuronal and adipocyte differentiation [6]. In recent years, a series of experiments have demonstrated that BMP-9 is more osteogenic than BMP-2 and is not antagonized by noggin, which is a BMP antagonist [7, 8]. BMP-9 was reported to have a positive role in promoting the MSCs differentiation through Smad signaling pathway [9]. However, the mechanism for BMP-9-induced osteogenic differentiation was unclear. Gene expression microarray used modern research method and has been used to explore the gene network and identify the potential target gene.

The purpose of our study was to compare the gene expression of BMP-9-induced osteogenic differentiation of MSCs in the GEO database. Subsequently, Gene Ontology (GO) and Kyoto Encyclopedia of Genes and Genomes (KEGG) enrichment pathway analyses were performed to identify the potential pathway of BMP-9-induced osteogenic differentiation of MSCs. Furthermore, protein-protein interaction (PPI) was performed to identify relevant genes and pathways and molecular mechanisms.

## Materials and methods

### Gene expression microarray data

The microarray data GSE48882 used in our study was downloaded from Gene Expression Omnibus (GEO, http://www.ncbi.nlm.nih.gov/geo/). GSE48882 was based on the Affymetrix GPL339 platform (Affymetrix Mouse Expression 430A Array; Affymetrix; Thermo Fisher Scientific, Inc., Waltham, MA, USA). The GSE48882 dataset contained six samples, including three GFP1-induced MSC samples and three BMP-9-induced MSC samples.

### Data processing and identification of DEGs

The original array data were performed background correction and quartile data normalization [10]. The raw data was downloaded and then put into the U-EDIT software to further analyses. Then, all of the differentially expressed genes (DEGs) between GFP-induced MSCs and BMP-9-induced MSCs were identified based on analyses GEO2R (https://www.ncbi.nlm.nih.gov/geo/geo2r/) and bioconductor package limma [11]. The DEGs between the GFP-induced MSC samples and three BMP-9-induced MSC samples were selected (*P* < 0.05).

### GO enrichment and KEGG pathway analysis of the DEGs

The DEGs was entered into the online software Database for Annotation, Visualization and Integrated Discovery (DAVID, https://david.ncifcrf.gov/) [12]. The DEGs were classified into three functional groups: molecular function (MF) group, biological process (BP) group, and cellular component (CC) group.

DEGs functional and signaling pathway enrichment were conducted using online websites of KEGG (http://www.genome.jp/) and *P* < 0.05 was considered to indicate a statistically significant difference [13].

### Construction of the PPI Network of DEGs

We downloaded the comprehensive interaction information of BMP-9-induced osteogenic differentiation of MSCs from the Search Tool for the Retrieval of Interacting Genes (STRING) database (http://www.string-db.org/) [14]. A combined score > 0.4 was identified as experimentally validated interactions. Then, we generate network and node file and analyzed using Cytoscape software (version 3.5.1; www.cytoscape.org). *P* < 0.05 was considered to be a statistically significant difference [15].

## Results

### Identification of DEGs

**Fig. 1:**
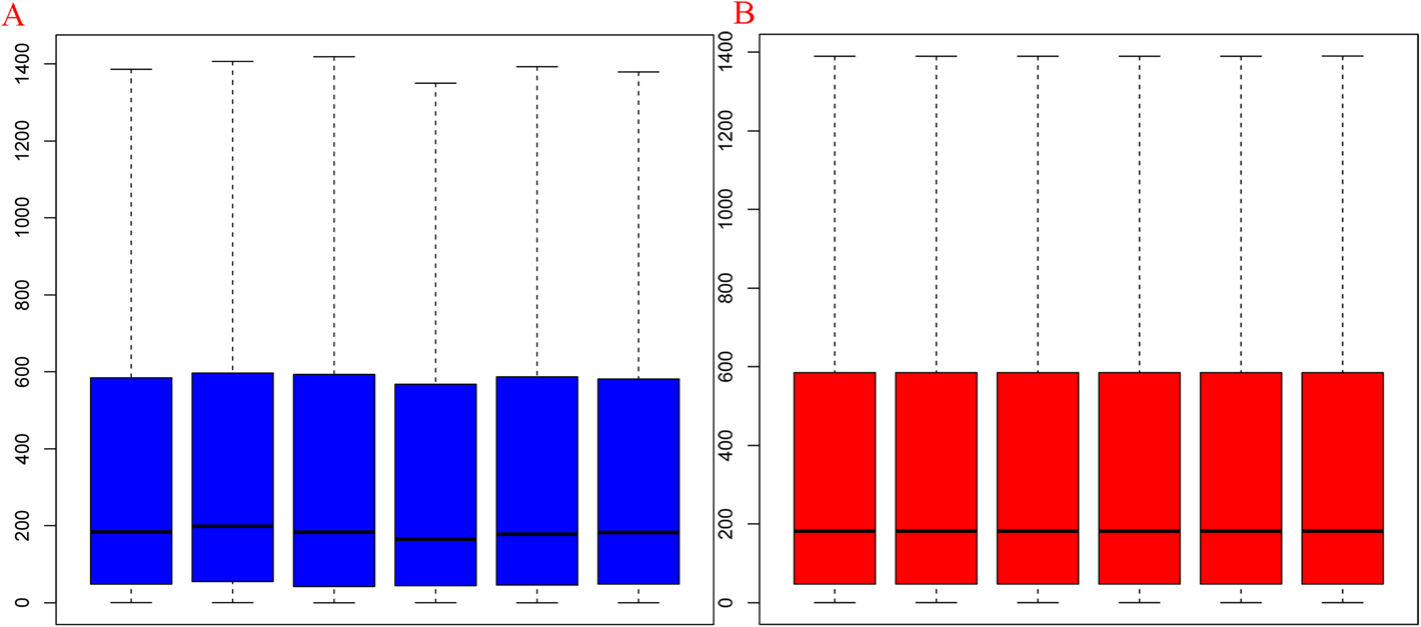
Box plots for expression data before normalization (**a**) and after normalization (**b**). The horizontal axis is the name of samples while the vertical axis stands for the values of expression. The black line in the cassette is the median of data in each group, which represents the degree of normalization. The black line in the right figure was almost on the same line, indicating an excellent degree of normalization

**Fig. 2:**
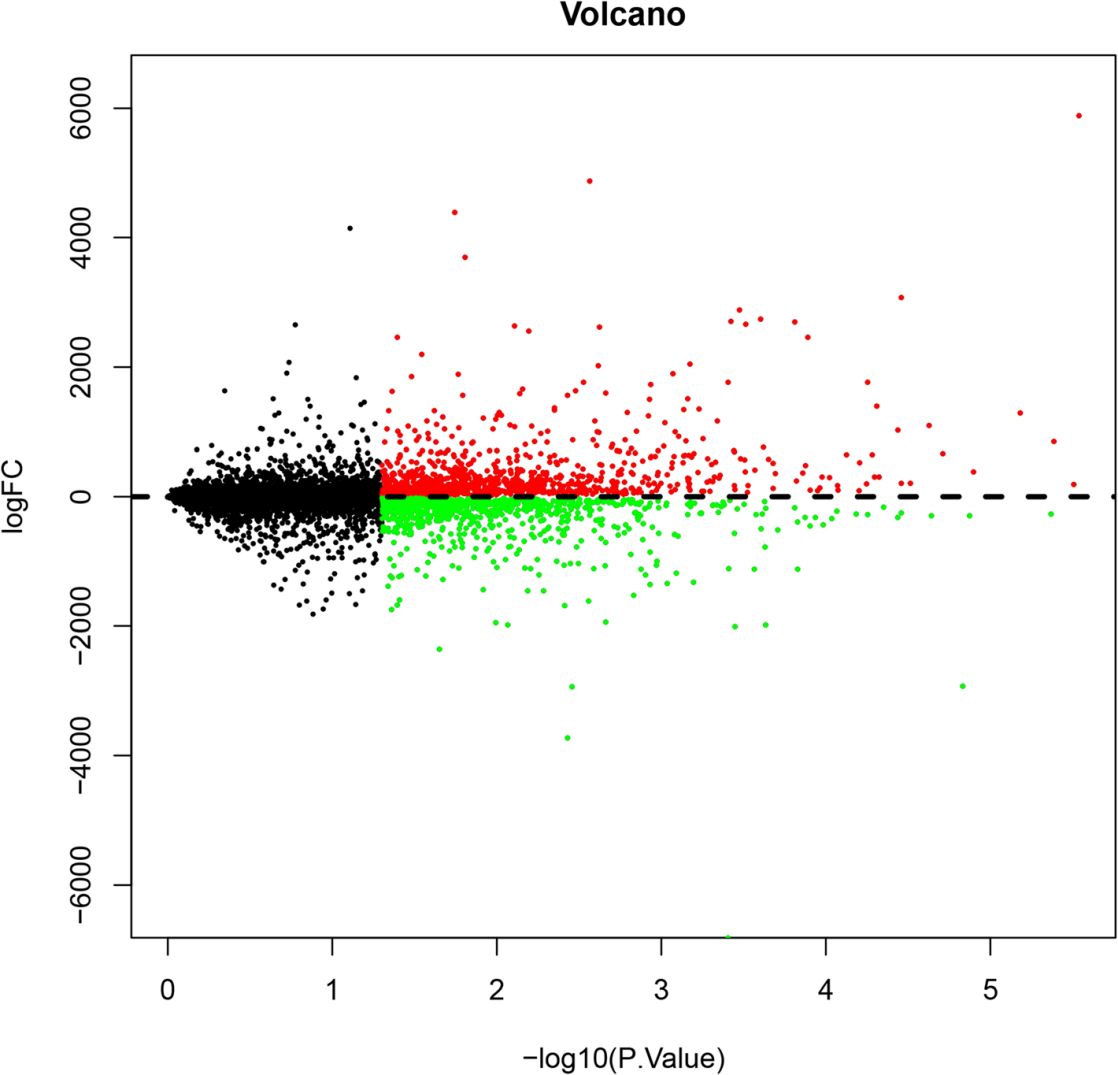
The volcano plot of differentially expressed genes. The abscissa is − log10 (P.Value) and the ordinates is logFC. The red dots stand for the up-expressed genes, green dots stand for down-expressed gene while the black dots represent genes not differentially expressed

**Fig. 3:**
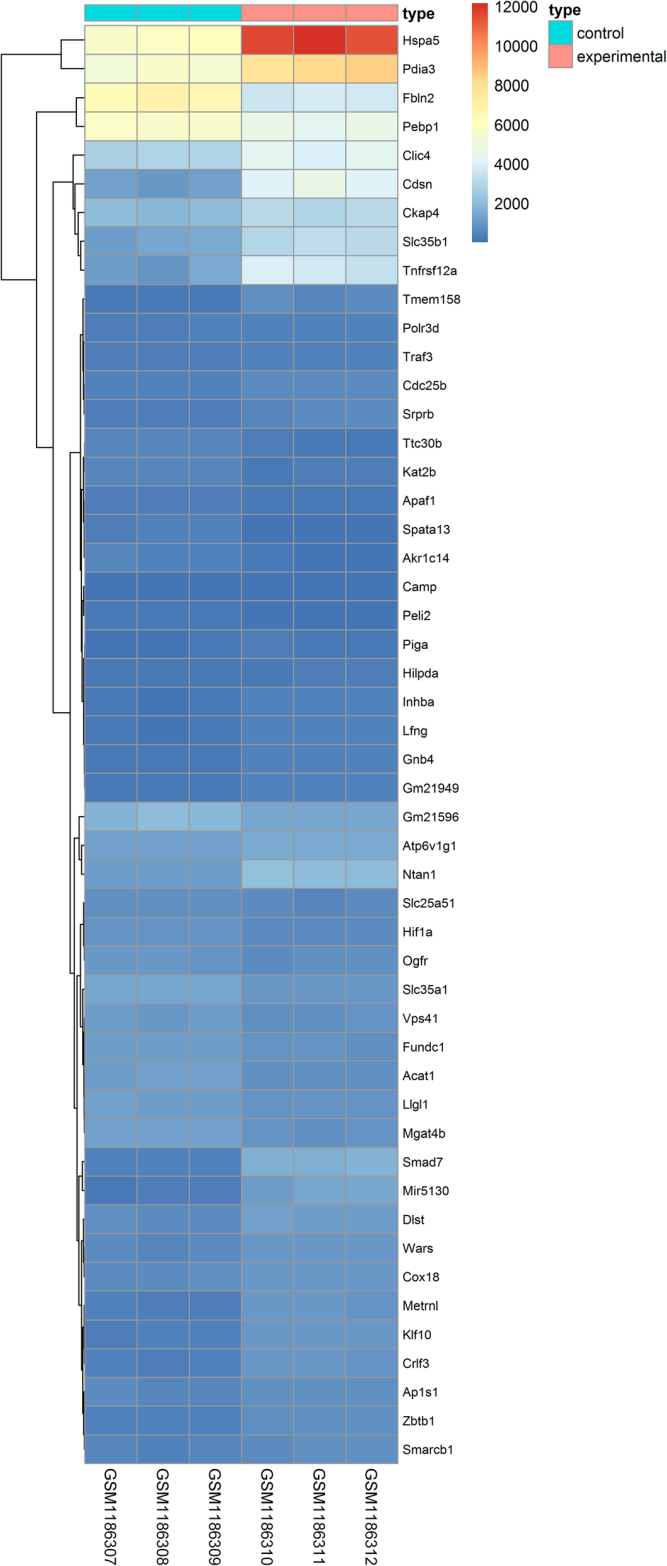
The heat map of the differentially expressed genes (top 50 upregulated and downregulated genes). Red, upregulation; blue, downregulation

*P* < 0.05, logFC (fold control) > 2.0 or logFC < − 2.0 was used in current study, and we listed the first tenth DEGs in Table 1. And the samples were normalized and results were shown in Fig. 1. Among the DEGs, 1029 genes (52.31%) and 938 genes (47.69%) were upregulated and downregulated, respectively. Then, we draw a volcano plot and can be seen in Fig. 2. The top 50 upregulated and downregulated DEGs were selected to generate the heatmap and is shown in Fig. 3.

**Table 1:** The differentially expressed genes (DEGs)

Gene	logFC	AveExpr	*t*	P.Value	adj.P.Val	*B*
Hspa5	5886.413	8805.691	24.55842	2.89E− 06	0.014437	− 4.41275
Atp6v1g1	192.8844	1430.236	24.16697	3.12E− 06	0.014437	− 4.41281
Ntan1	849.4093	1610.547	22.81636	4.11E− 06	0.014437	− 4.41304
Ttc30b	− 269.874	376.4807	− 22.6356	4.27E− 06	0.014437	− 4.41307
Smad7	1291.794	1054.716	20.67191	6.60E− 06	0.017846	− 4.4135
Wars	382.1337	817.2169	18.04368	1.27E− 05	0.024944	− 4.41429
Hif1a	− 297.61	791.8699	− 17.8299	1.34E− 05	0.024944	− 4.41437
Fbln2	− 2928.99	5171.091	− 17.4703	1.48E− 05	0.024944	− 4.41452
Klf10	665.8488	694.2806	16.49257	1.94E− 05	0.029027	− 4.41495
Kat2b	− 295.886	399.0065	− 15.9344	2.29E− 05	0.029027	− 4.41523

### GO term enrichment analysis

**Fig. 4:**
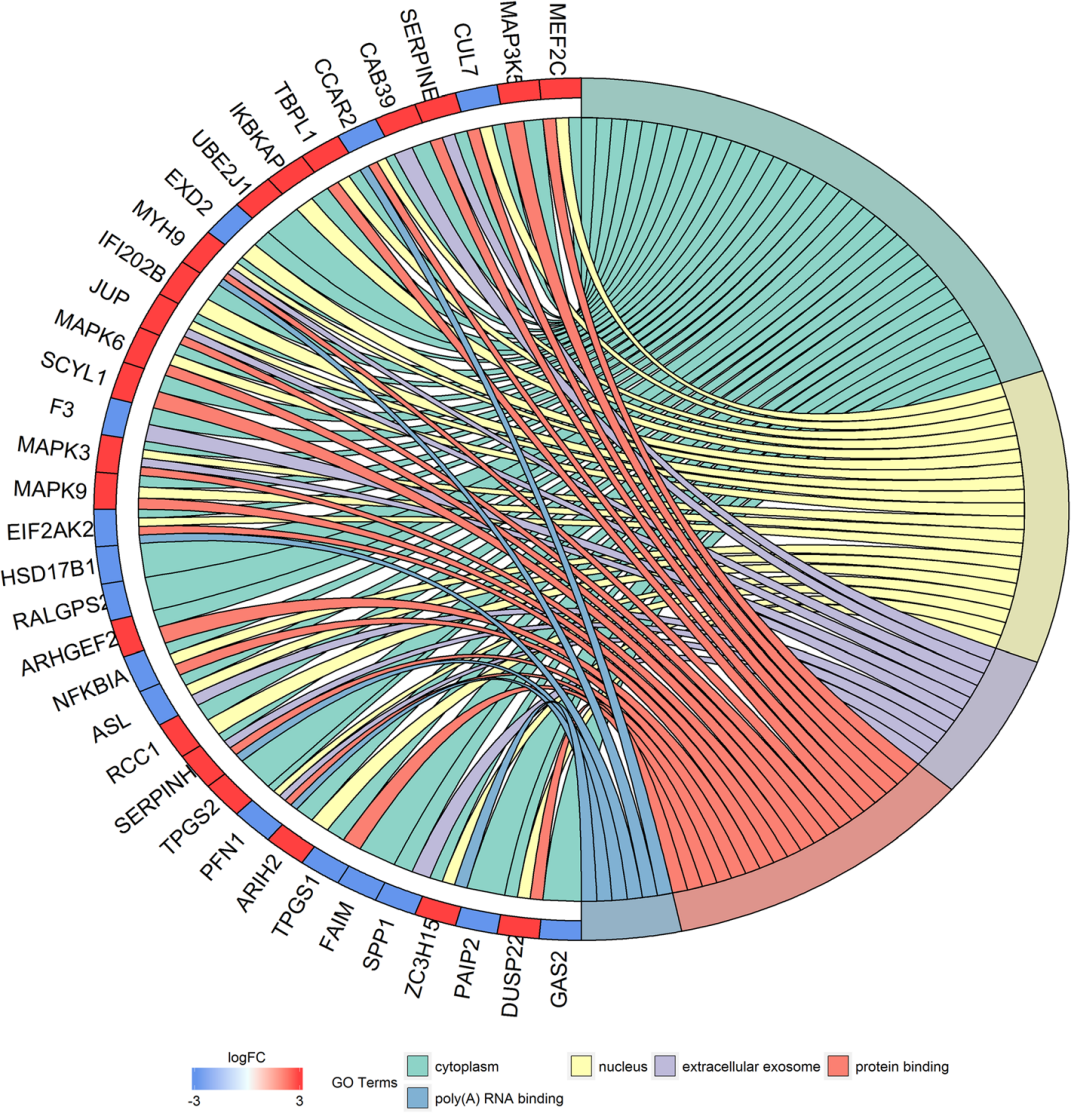
Go term enrichment analysis of the different genes

DAVID was used to carry out a gene ontology (GO) function enrichment for DEGs. Results show that the DEGs were significantly enriched in CC, including cytoplasm, nucleus, and extracellular exosome (Fig. 4). For MF, the DEGs were enriched in protein binding and poly(A) RNA binding (Fig. 4).

### KEGG pathway analysis

**Fig. 5:**
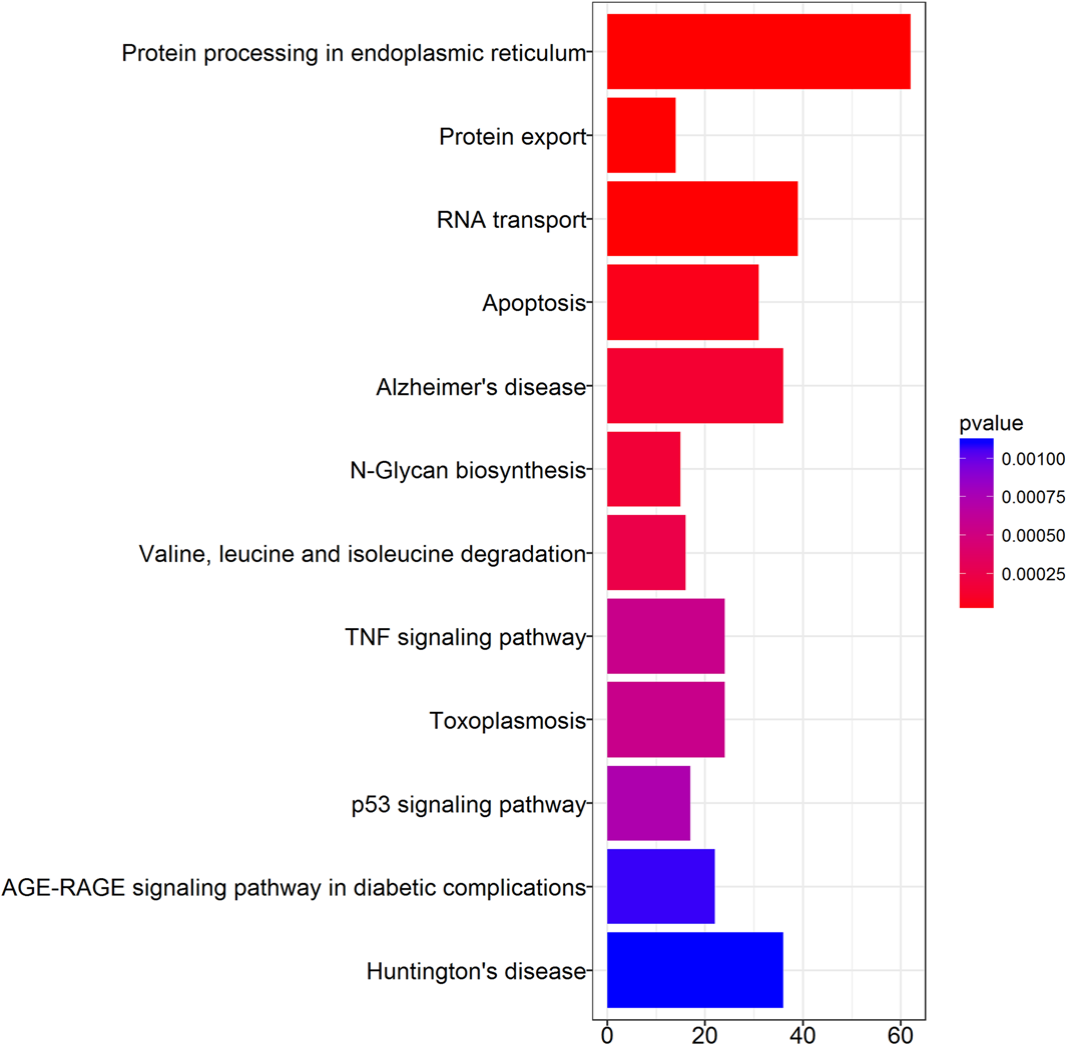
Barplot analysis of the Kegg pathway

**Fig. 6:**
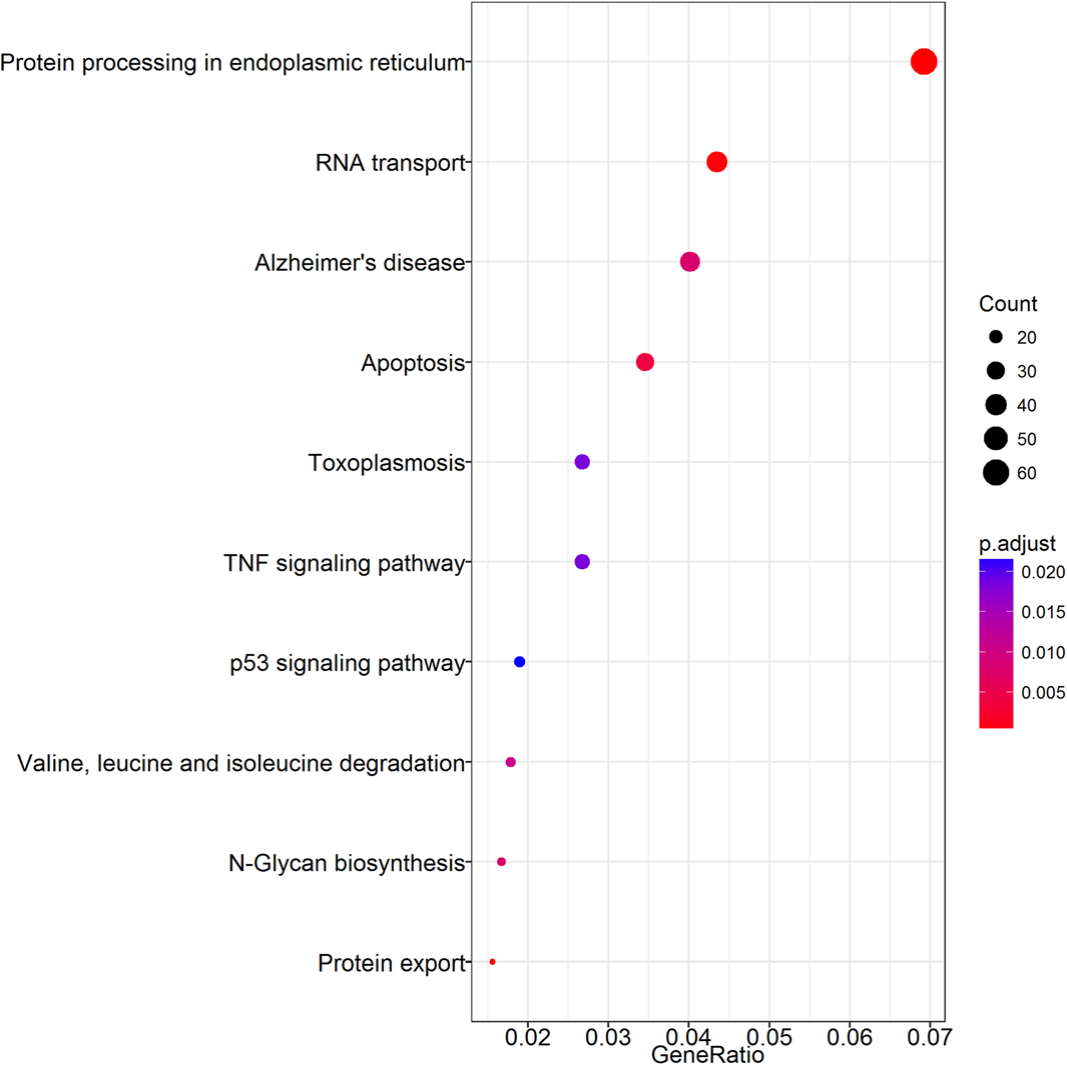
Dotplot analysis of the Kegg pathway

Results show that the DEGs were enriched in protein processing in endoplasmic reticulum, protein export, RNA transport, apoptosis, Alzheimer’s disease, N-Glycan biosynthesis, Valine, leucine and isoleucine degradation, TNF signaling pathway, Toxoplasmosis, p53 signaling pathway, and AGE-RACE signaling pathway in diabetic complications (Figs. 5 and 6).

### PPI network of the DEGs and core genes in the PPI network

**Fig. 7:**
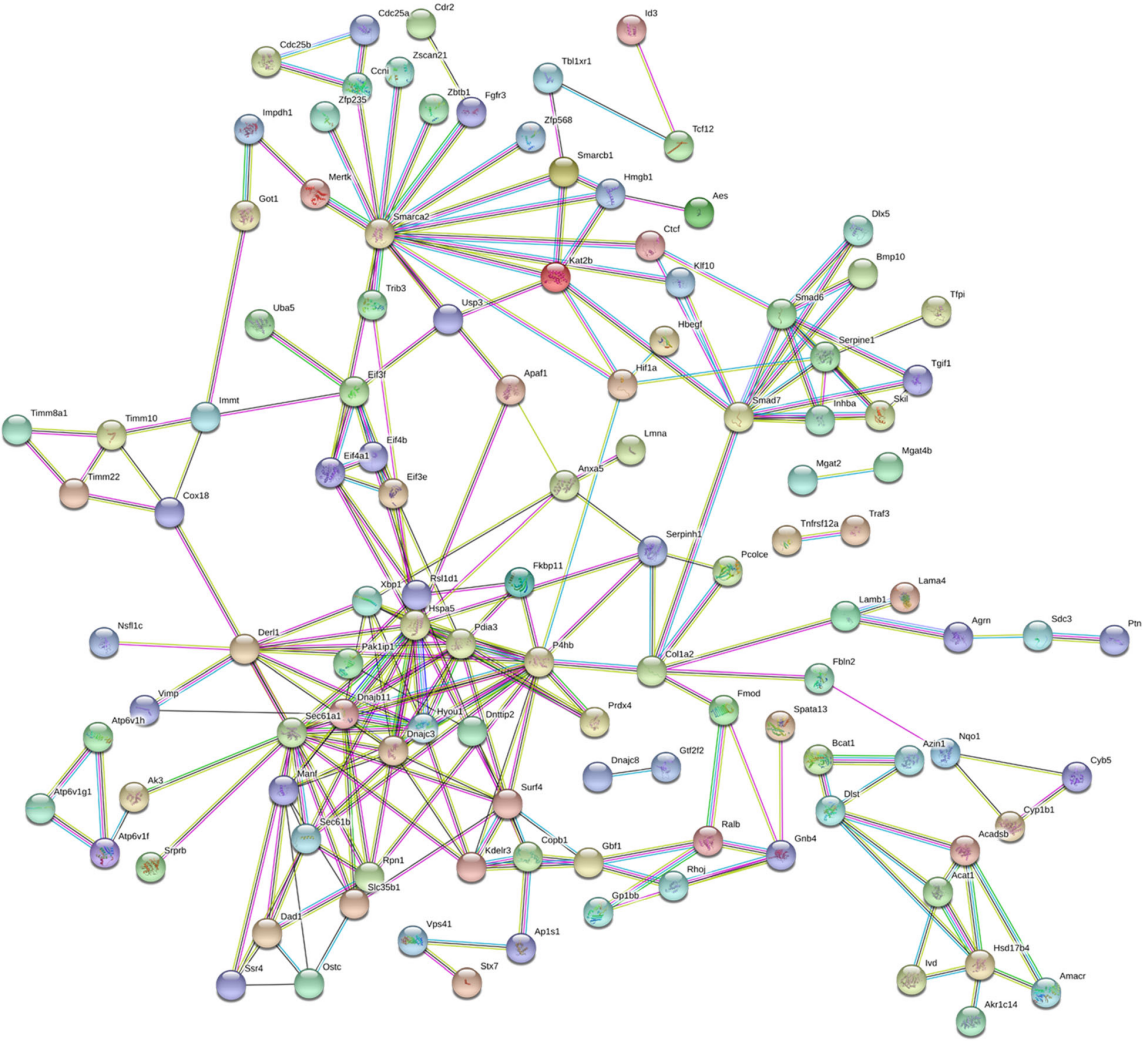
PPI network of BMP-9 induced osteogenic MSCs genes. The circle stands for genes and the line indicates the interactions among genes. The interior of the circle represents the structure of proteins. The color of the line provides evidence of the different interactions among proteins. (A red line indicates the presence of fusion evidence; a green line, neighborhood evidence; a blue line, concurrence evidence; a purple line, experimental evidence; a yellow line, text mining evidence; a light blue line, database evidence; a black line, coexpression evidence)

**Fig. 8:**
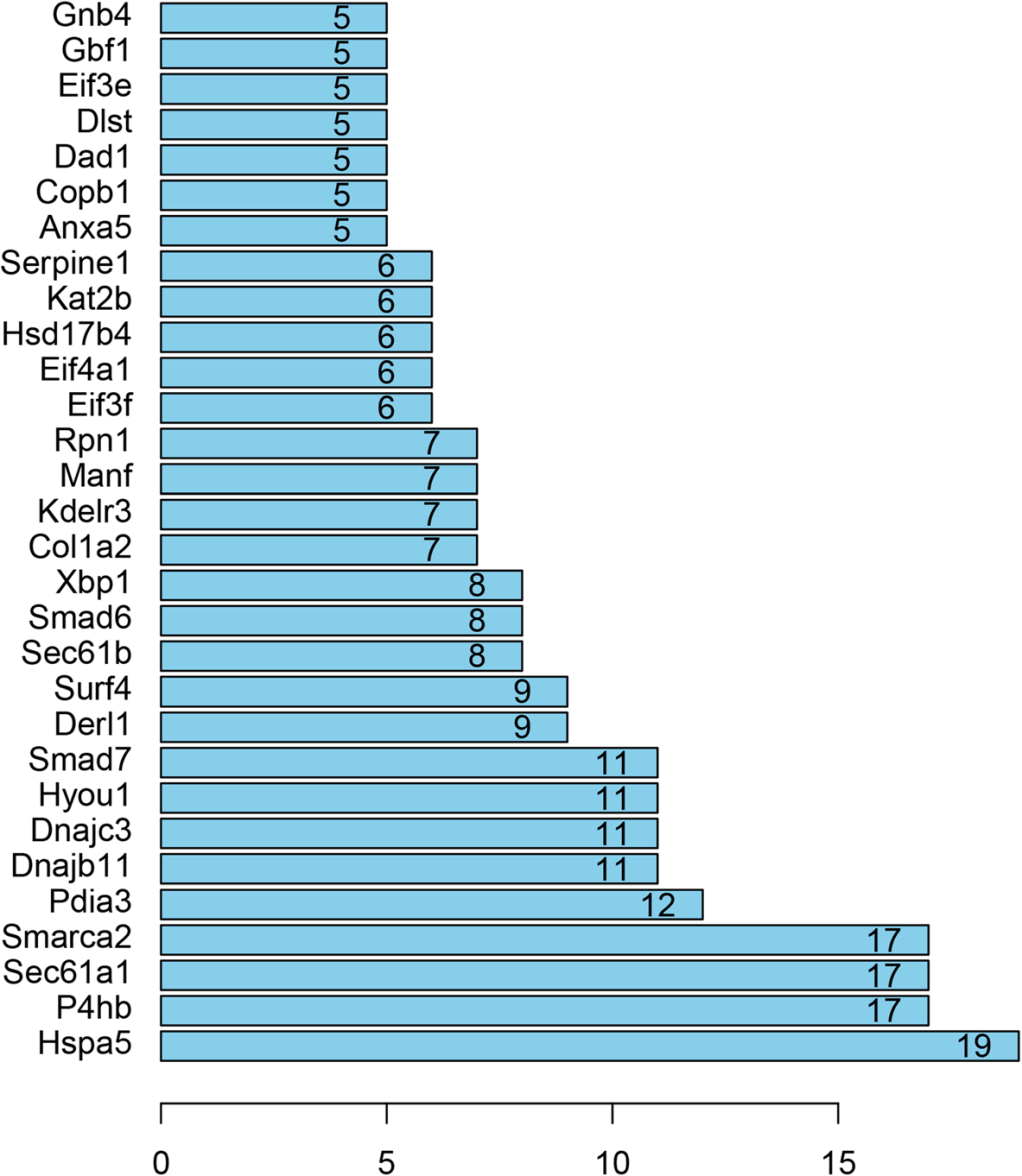
Histogram of numbers of genes adjacent to interaction network

**Fig. 9:**
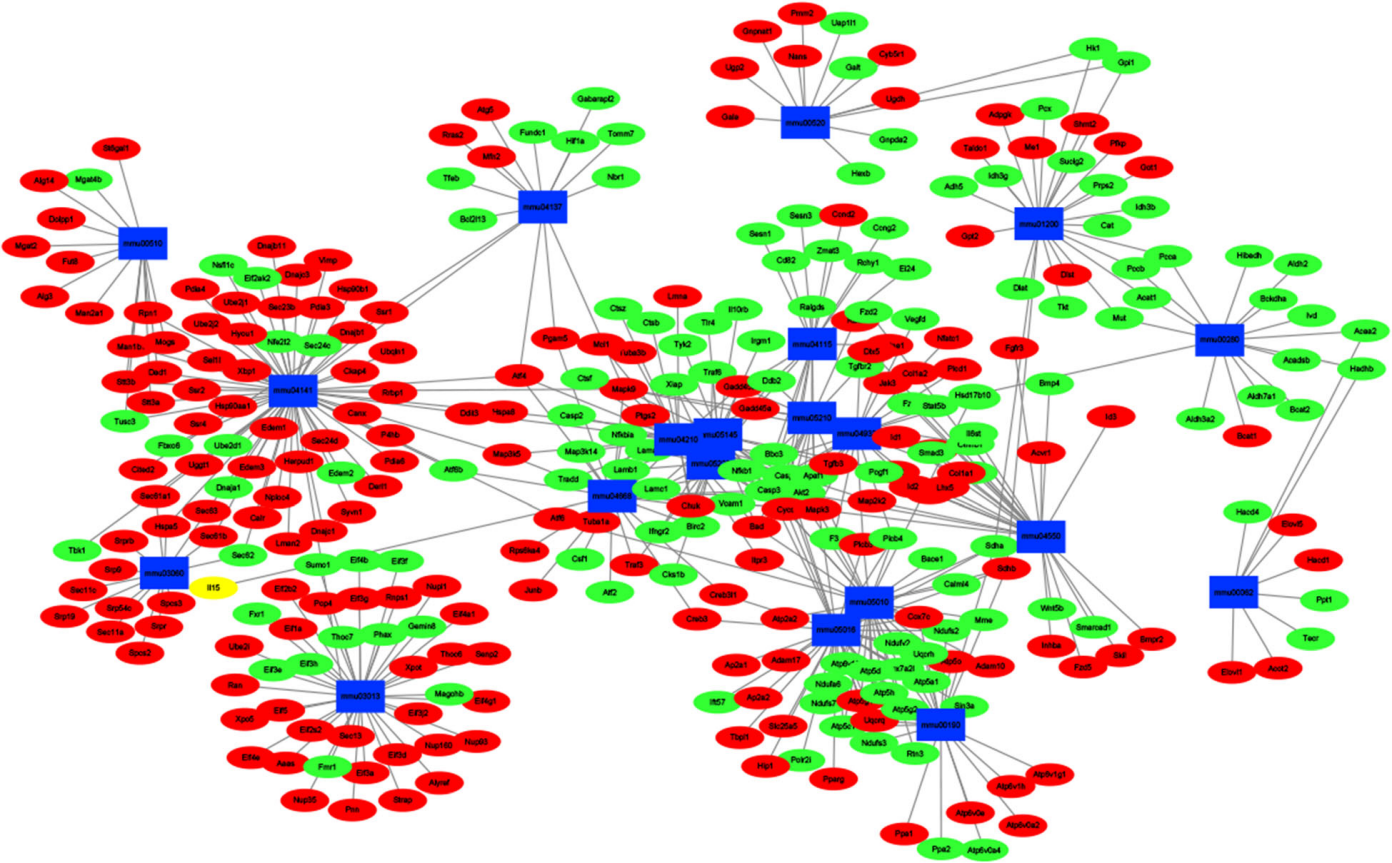
Interaction network of the proteins

As shown in Fig. 7, the PPI network contained 197 nodes and 272 edges. The top 10 high-degree hub nodes included Hspa5, P4hb, Sec61a1, Smarca2, Pdia3, Dnajc3, Hyou1, Smad7, Derl1, and Surf4 (Fig. 8). Further analyses of the DEGs, Hspa5’s node degree score was 108 and identified as the highest node degree (Figs. 8 and 9).

## Discussion

In the clinical application of rhBMP-2, adverse events such as tissue inflammation and bone resorption perplexed the physician [16–18]. Therefore, there is a crying need for the development of an equivalent effective and more safe bone regenerative therapies for bone regeneration. Recently, we attached importance to the rhBMP-9 since rhBMP-9 possesses different properties than rhBMP-2 [19–21]. Its precursor protein shares 50–55% amino acid sequence identity with BMP 2, 4, 5, 6, and 7. However, the present knowledge of the molecular mechanism of BMP-9-induced osteogenic differentiation of MSCs remains insufficient.

The present study performed a comprehensive analysis and built a gene interaction network based on the gene expression profiles (GSE48882) comprising three GFP-MSC samples and three BMP-9-MSC samples. The results of the analysis demonstrated that 1967 genes were identified as DEGs. Moreover, GO and KEGG pathway analyses were performed to find the interactions of DEGs. Combining with the PPI network, key potential genes and pathways may be associated with BMP-9-induced osteogenic differentiation of MSCs.

The results of GO analyses indicated cytoplasm, nucleus and extracellular exosome, protein binding and poly(A) and RNA binding. Nakamura et al. [22] revealed that BMP-9 provided comparable new bone formation and with less adipose tissues compared to BMP-2 in calvarial critical-size defects model. Recently, Sreekumar et al. [23] found that rhBMP9 induced the highest levels of osteoblast activity when compared to the clinically utilized rhBMP2 and rhBMP7. Compared with rhBMP2 and rhBMP7, rhBMP9 significantly increased osteogenic activity (ALP activity and Smad nuclear translocation).

Lin et al. [24] also found that BMP9-induced osteogenic differentiation through inhibition of the Wnt/β-catenin and P38 pathway in MSCs. And adding Wnt antagonist Dickkopf-1 (Dkk1) or P38 inhibitor SB203580 could negatively regulate BMP9-induced osteogenic differentiation. In current study, gene array indicated that BMP-9 has no effect on the Wnt/β-catenin pathway.

Chen et al. [25] found that BMP-9 induce osteogenic differentiation of MSCs through Smad pathway. When there was an overexpression of the BMP-9 in MSCs, Smad 1, Smad 7, and Smad 9 was significantly increased than controls. And insulin-like growth factor (IGF-1) could enhance BMP9-induced osteogenic differentiation in MSCs through Smad signaling pathway. The above result was similar with our results and found that BMP-9 could also increase the expression of Smad7. PPI network revealed that Smad 7 ranked the eighth of the high-degree hub nodes. Daigang et al. [26] found that BMP-9 stimulates the phosphorylation of Smad1/5/8 at a dose response manner. What is more, BMP-9 could significantly increase osteoblast differentiation genes such as Runx2, osteocalcin, and ALP.

Hspa5 was the heat shock protein and plays an important role in protein folding and transport processes. Hspa5 plays a role in facilitating the assembly of multimeric protein complexes inside the endoplasmic reticulum. And thus, BMP-9 may through protein folding and transport and promote osteogenic differentiation of MSCs. Sharff et al. [27] revealed that Hey1, through its interplay with Runx2, may play an important role in regulating BMP9-induced osteoblast lineage differentiation of MSCs. Zhou et al. [28] revealed that BMP9 reduces bone loss in ovariectomized mice by dual regulation of bone remodeling. BMP9 exerts on bone remodeling by promoting bone anabolic activity and inhibiting osteoclast differentiation in ovariectomy (OVX) mice. Hey1 and Hspa5 interact with each other and promote the expression of MSCs. And from the PPI analysis, we found that HIF-1a also plays a central role in the osteogenic differentiation of MSCs. As we know, HIF-1a can stimulate the VEGF and stimulate the angiogenesis [29–31].

Several limitations existed in our study: (1) we did not verify the key gene and pathways through PCR and Western-blot method; (2) key miRNA and lncRNA were not further analyzed and should be performed in future research; and (3) optimal dose of BMP-9 was not explored in this gene expression microarray. Future studies should be focused on the specific mechanism BMP-9-induced osteogenic differentiation of MSCs. What is more, administration with small interference RNA to further confirms our conclusion.

## Conclusion

In conclusion, our study provides a comprehensive bioinformatics analysis of key genes and pathways of BMP-9 induced osteogenic differentiation of MSCs. Their related GO terms found that cytoplasm and nucleus may play important roles in BMP-9-induced osteogenic differentiation of MSCs and have the potential to be used as an potential agent for MSCs osteogenic differentiation. Further genetic and experimental studies with larger sample size are needed to confirm our results.

## Data Availability

We state that the data will not be shared since all the raw data are present in the figures included in the article.

## Abbreviations

DEGs: Differentially expressed genes
MSCs: Mesenchymal stem cells
GEO: Gene Expression Omnibus
KEGG: Kyoto Encyclopedia of Genes and Genomes
PPI: Protein-protein interaction
BMPs: Bone morphogenetic proteins
TGF-β: Transforming growth factor-β
GO: Gene Ontology
DAVID: Database for Annotation, Visualization and Integrated Discovery
MF: Molecular function
BP: Biological process
CC: Cellular component
STRING: Search Tool for the Retrieval of Interacting Genes
Dkk1: Dickkopf-1
IGF-1: Insulin-like growth factor
